# Novel Pathogenic Variant in TGFBR2 Confirmed by Molecular Modeling Is a Rare Cause of Loeys-Dietz Syndrome

**DOI:** 10.1155/2017/7263780

**Published:** 2017-01-09

**Authors:** Michael T. Zimmermann, Raul A. Urrutia, Patrick R. Blackburn, Margot A. Cousin, Nicole J. Boczek, Eric W. Klee, Colleen Macmurdo, Paldeep S. Atwal

**Affiliations:** ^1^Division of Biomedical Statistics and Informatics, Department of Health Sciences Research, Mayo Clinic, Rochester, MN, USA; ^2^Laboratory of Epigenetics and Chromatin Dynamics, Departments of Biochemistry and Molecular Biology, Biophysics, and Medicine, Mayo Clinic, Rochester, MN, USA; ^3^Department of Clinical Genomics, Center for Individualized Medicine, Mayo Clinic, Jacksonville, FL, USA; ^4^Center for Individualized Medicine, Department of Clinical Genomics, Mayo Clinic, Rochester, MN, USA; ^5^Division of Medical Genetics, Department of Pediatrics, Stanford University, Stanford, CA, USA

## Abstract

Loeys-Dietz syndrome (LDS) is a connective tissue disorder characterized by vascular findings of aneurysm and/or dissection of cerebral, thoracic, or abdominal arteries and skeletal findings. We report a case of a novel pathogenic variant in* TGFBR2* and phenotype consistent with classic LDS. The proband was a 10-year-old presenting to the genetics clinic with an enlarged aortic root (*Z*-scores 5-6), pectus excavatum, and congenital contractures of the right 2nd and 3rd digit. Molecular testing of* TGFBR2* was sent to a commercial laboratory and demonstrated a novel, likely pathogenic, variant in exon 4, c.1061T>C, p.(L354P). Molecular modeling reveals alteration of local protein structure as a result of this pathogenic variant. This pathogenic variant has not been previously reported in LDS and thus expands the pathogenic variant spectrum of this condition.

## 1. Background

Loeys-Dietz syndrome (LDS) is a connective tissue disorder characterized by vascular findings of aneurysm and/or dissection of cerebral, thoracic, or abdominal arteries and skeletal findings comprising pectus abnormality, scoliosis, arachnodactyly, hypermobile joints, congenital contractures of digits, and talipes equinovarus [1, 2]. In addition, craniofacial findings including hypertelorism, bifid uvula, cleft palate, and craniosynostosis are commonly observed in patients affected by LDS. Intellect is generally normal and typically patients develop arterial aneurysms with death resulting from a ruptured arterial aneurysm occurring in the third decade of life if left undiagnosed [2, 3].* TGFBR1 *and* TGFBR2* pathogenic variants were initially shown to cause LDS, with subsequent pathogenic variants identified in* SMAD3*,* TGFB2, and TGFB3*. We report a case of a novel pathogenic variant in* TGFBR2* and phenotype consistent with LDS [4] and demonstrate pathogenic effects on the downstream protein structure based on novel molecular modeling techniques.

## 2. Presentation

The proband was a 10-year-old presenting to the genetics clinic with an enlarged aortic root (*Z*-score 5-6), pectus excavatum, and congenital contractures of the right 2nd and 3rd digit. His pediatrician originally referred him to a local cardiologist for a suspected connective tissue disorder. He was born at term to a 19-year-old G2P2 mother and 31-year-old father. Birth weight and length were normal; however, he was noted to have contractures of the right 2nd and 3rd digit. Other medical history includes a mild restrictive lung disease that was felt to be secondary to his moderately severe pectus excavatum. His development had been normal and he was in mainstream education. A three-generation family pedigree did not reveal any consanguinity. Both parents were unaffected. Physical examination demonstrated head circumference of 50 cm (50th percentile), height of 150 cm (84th percentile), weight of 33.2 kg (42nd percentile), arm span of 156 cm, arm span/height ratio of 1.04, and upper to lower segment ratio of 0.923. He was normocephalic and hyperteloric (inner canthal distance 4 cm, >97th percentile; outer canthal distance 10 cm, >97th percentile; interpupillary distance 6.5 cm, >97th percentile). He had nasal asymmetry and a bifid uvula. There were marked pectus excavatum, mild scoliosis, shoulder hyperextensibility, negative wrist and thumb sign, no arachnodactyly, pes planus, and thin translucent skin over anterior chest and arms with evidence of easy bruising. Echocardiogram demonstrated normal valves with a severely dilated aortic root between 5-6* Z*-scores. He was prescribed losartan 1 mg/kg/day managed by a cardiologist.

Molecular testing of a gene panel including* TGFBR1, TGFBR2, SMAD3*,* TGFB2, and TGFB3* was performed using standard techniques via CLIA/CAP certified commercial laboratory and demonstrated a novel likely pathogenic variant in exon 4 of TGFBR2, denoted by c.1061T>C, p.(L354P). The variant is in a highly conserved region of DNA (13/13 species). The change occurs in the serine-threonine kinase domain of the protein. Parental testing performed at the same laboratory revealed this was a de novo change.

Additional findings include pulmonary function tests confirming a restrictive lung defect, likely related to the pectus deformity, not reversible by bronchodilators, for which he is followed up by pulmonology. An ophthalmological exam did not find any evidence of myopia or subluxation. An MRI/MRA head to pelvis was performed which demonstrated dolichoectasia of the internal carotid and left vertebral arteries, tortuosity of vessels within the chest, particularly vessels arising from the aortic arch, high-grade stenosis at the origins of the right common carotid and right subclavian arteries and proximal left common carotid artery, marked pectus deformity (Haller index of 13.4) causing compression of the right atrium and leftward shift of heart, and lastly a mildly tortuous left renal artery. The clinical features found in our patient were consistent with the diagnosis of LDS.

Interestingly, the patient was followed up by the allergy clinic for a history of idiopathic urticaria. The patient was placed on cetirizine and his symptoms resolved. TGFBR pathogenic variants have been shown to increase the predisposition for allergic disease (including asthma, eczema, and allergic rhinitis) in patients with LDS because of their role in directing immune responses to mucosal antigens [5]. A 2013 study suggested that treatment with losartan could modify allergic disease in patients by reducing excessive TGFB signaling in lymphocytes [5].

## 3. Molecular Modeling

**Figure 1:**
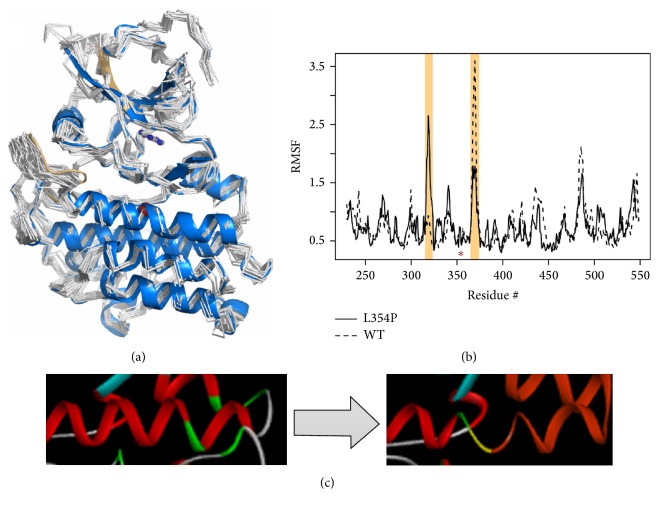
Molecular modeling of the L354P mutation reveals alteration of local structure leading to changes in flexibility. (a) The* TGFBR2* kinase domain structure is shown in a blue cartoon representation with amino acid position 354 marked by a red sphere and an adenosine placed in the ATP binding pocket shown as ball-and-stick. Representatives from the beginning of our MD simulation are shown as white backbone trace. (b) Root Mean Squared Fluctuations (RMSF) capture the flexibility of the protein and are plotted for the mutant and wt simulations. Regions with largest difference are highlighted (colored similarly in (a)) and position 354 is marked with an asterisk. (c) During our simulation, the local helical structure around L354P is destabilized and unwinds, pushing the rest of the helix into an altered conformation.

We developed a homology-based model for the* TGFBR2* kinase domain based on the experimentally solved structure of Activin Receptor IIA (see Figure 1) using available structural information for other members of this family of serine/threonine kinases. Ramachandran plots identified 96.5% of the residues in the allowable regions, reflecting the high quality of our model. Subsequently, we developed a model for the L354P variant by in silico site directed mutagenesis. These models were energy minimized, were heated to room temperature, and underwent short molecular dynamics simulation.

Molecular dynamics (MD) simulations were performed for our WT and L354P models using the CHARMm all-atom force-field [6] and a 2 fs time step. A distance-dependent implicit environment model, as implemented in Discovery Studio [7], was used with a dielectric constant of 80 and a pH of 7.4. Each model was energy minimized for 5000 steps using steepest decent followed by 5000 steps of conjugate gradient and the SHAKE [8] procedure. Each system was independently heated to 300 K and equilibrated, followed by generation of 10 ns production simulations. Analyses were performed in the R programming language [9], leveraging the bio3d [10] package version 2.2.4. Molecular visualizations were generated using Discovery Studio and PyMol [11].

These simulations revealed loss of local secondary structure around L354P as well as resulting shifts in molecular flexibility. Schematic representation of the protein molecular model is shown in Figure 1. The L354P variant introduces a kink within helix E, a secondary structural feature located underneath the floor of the ATP binding site. We posit that the L354P pathogenic variant may change the dynamics or molecular flexibility of the protein, thereby impacting other key functional regions within the protein.

## 4. Discussion

LDS is a childhood onset connective tissue disorder characterized by distinctive vascular, anomalies, and molecular abnormalities. Pathogenic variants in all 5 genes that cause LDS,* TGFBR1, TGFBR2, TGFB2, TGFB3, and SMAD3* result in loss of wild-type function of the protein product. The novel pathogenic variant identified in our patient is located in the intracellular serine-threonine kinase domain, which is where the majority of pathogenic variants are found. According to published data, most pathogenic variants in TGFBR2 are missense, and only a handful of nonsense pathogenic variants have been reported. It has been reported previously that the phenotype in LDS results due to excess activation and signaling by the TGF-*β* growth factor [3, 12]. Molecular confirmation is highly warranted as it can confirm a diagnosis suspected on clinical grounds and additionally it is important to identify the underlying molecular mutation in cases such as ours as it can be useful to (1) inform management, (2) confirm the diagnosis, and (3) provide actionable information for family planning purposes. Additionally, we demonstrate a novel molecular modeling technique which can be useful for determining pathogenicity of newly identified variants. This technique is applicable to many different genes and proteins and not specific to* TGFBR2*. In summary, we describe a patient with LDS and a novel* TGFBR2* pathogenic variant, previously undescribed as causal for LDS, and thus expand the mutation spectrum of this condition.

## Abbreviations

LDS: Loeys-Dietz syndrome.
